# Glucose dynamics in glycogen storage disease type IXa with novel *PHKA2* variants: insights from our experience and a comprehensive review of the disease spectrum

**DOI:** 10.1007/s42000-025-00712-9

**Published:** 2025-08-28

**Authors:** Federico Baronio, Giacomo Biasucci, Egidio Candela, Maria Giulia Regazzi, Valeria Di Natale, Rita Ortolano, Marcello Lanari

**Affiliations:** 1 Pediatric Unit, IRCCS Azienda Ospedaliero-Universitaria di Bologna, via Massarenti 9, Bologna, Italy; 2 Pediatrics and Neonatology Unit, Guglielmo da Saliceto Hospital, Piacenza, 29121 Italy; 3 Department of Medical and Surgical Sciences, Alma Mater Studiorum, University of Bologna, via Massarenti 11, Bologna, Italy; 4 Department of Medicine and Surgery, University of Parma, Parma, 43126 Italy; 5 Clinical Nutrition and Metabolism Unit- IRCCS Azienda Ospedaliero- Universitaria di Bologna, via Massarenti 9, Bologna, Italy

**Keywords:** Glycogen storage disease IX, GSD IX, PHKA2, Ketotic hypoglycemia, Pediatric endocrinology, Pediatric metabolic disease, Glycosade, Uncooked cornstarch, Extended-release cornstarch

## Abstract

**Background:**

Glycogen storage disease type IXa (GSD IXa), caused by phosphorylase kinase mutations, primarily PHKA2, presents variably from mild hepatomegaly to severe liver dysfunction or isolated ketotic hypoglycemia. Its phenotypic overlap with other metabolic disorders complicates diagnosis, requiring genetic confirmation.

**Methods:**

We retrospectively analyzed clinical, biochemical, genetic, and radiological data from patients affected by GSD IXa diagnosed at our regional metabolic disease center in Bologna (Emilia-Romagna, Italy) over recent decades and we reviewed the relevant scientific literature on the pathology.

**Results:**

We report three patients with recurrent symptomatic ketotic hypoglycemia affected by PHKA2 variants of uncertain significance (VUS) and review the literature concerning GSD IXa.

**Conclusion:**

GSD IXa should be considered in the differential diagnosis even when persistent ketotic hypoglycemia is the sole presenting feature, underscoring the critical importance of clinical suspicion in such cases. Improved genetic testing has refined diagnosis, even in milder cases without hepatomegaly, while dietary management with uncooked cornstarch (CS) and extended-release cornstarch (ER-CS) effectively prevents complications and maintains good glycemic control.

## Introduction

Glycogen storage diseases (GSDs) are inborn metabolic disorders characterized by alterations of enzymes implicated in the synthesis or degradation of glycogen [1]. Within this category, glycogen storage disease type IX, or GSD type IX (OMIM 306000), is due to a phosphorylase kinase (PhK) defect. This enzyme regulates the conversion of glycogen to glucose [2]. PhK is a tetramer composed of four different subunits (α, β, γ, and δ), each subunit having tissue-specific isoforms codified by multiple genes [3]. The α-, β-, and γ-subunits have liver-specific isoforms encoded by, respectively, PHKA2, PHKB, and PHKG2 [3]. GSD type IXa (GSD IXa) is associated with mutations in PHKA2 and transmitted as an X-recessive trait; it constitutes about 75% of total cases of GSD IX, the most frequent type of GSD, with a prevalence of 1:100,000 [4].

GSD IX is an insidious disease and its diagnosis is complex because of the overlap of clinical phenotypes with other congenital diseases, such as other glycogenoses (GSD III, VI) or diseases related to carbohydrate metabolism (glucose-6-phosphatase deficiency and fructose-1,6-bisphosphatase deficiency). Classically, the clinical features commonly associated with GSD type IX are a characteristic “doll face,” short stature, mild to gross motor delay, and progressive liver disease often advancing to liver cirrhosis [5]. However, milder or attenuated forms of GSD IXa have been reported, mainly characterized by hepatomegaly along with elevated liver enzymes. Hypoglycemia is not a key feature of this disease since gluconeogenesis and fatty acid oxidation are preserved [6]. Therefore, lactic acid and blood glucose concentrations are often regular [7].

However, over the years, other phenotypes of GSD IX have emerged. GSD IX γ2 appears to be the most severe phenotype, persisting with age, with progressive liver involvement that can lead to cirrhosis and hepatocellular carcinoma [5]. Genitope GSD IX β, besides hepatic involvement, appears most frequently associated with delayed growth [8].

Recently, a cohort of patients with PHKA2 mutations was described as isolated ketotic hypoglycemia without hepatomegaly [9].

We report three patients with recurrent symptomatic ketotic hypoglycemia affected by PHKA2 variants of uncertain significance (VUS) and review the literature concerning GSD IXa, with a focus on our national case history.

## Materials and methods

We retrospectively reviewed clinical charts and collected the clinical, biochemical, genetic, and radiological data of all the patients diagnosed with hepatic GSD IXa at our regional reference center for newborn screening and metabolic diseases. This cohort includes all known cases identified and followed at our institution over the past decades, providing a complete overview of the GSD IXa population managed at our center.

Clinical and biochemical data were collected during the first visit and the follow-up. Patients were routinely evaluated at our center every 3–6 months. During follow-up, height (cm) was measured with the Harpenden stadiometer, which has a precision of 0.1 cm, and weight (kg) was obtained using a steelyard scale, with an accuracy of 0.1 kg. Stature and weight were then compared with the reference growth curves of Italian percentiles using SIEDP Growth 4.0^®^ by Eli Lilly [10].

The case reports were described according to the international CARE guidelines [11]. In two out of three cases, patients were identified by an initial outpatient visit, with admission to our center and metabolic and genetic investigations. We collected blood tests to assess organ involvement, focusing on blood count, coagulation, glucose, lipid profile (total cholesterol, HDL, LDL, and triglycerides), ALT, AST, uric acid, GGT, CPK, LD, basal insulin, IGF-1, TSH and FT4, and blood gas analysis.

Diagnostic confirmation was performed using an NGS gene panel for hypoglycemia. Individual variants were characterized using coding impact, through Varsome [12] and, for the interpretation of sequence variants, using the American College of Medical Genetics and Genomics and the Association for Molecular Pathology (ACMG/AMP) guidelines [13].

We reviewed the published data on GSD IXa by performing a comprehensive search through the Pubmed, MEDLINE, and Scopus platforms. These databases were queried using individual keywords and MeSH terms. Keywords were mixed in different combinations using the Boolean operators “AND” or “OR” as appropriate and database-related filters to maximize the identification of articles. The entry MeSH terms for the PubMed search were “Glycogen Storage Disease Type IX” and “Phosphorylase Kinase Deficiency,” “Phk Deficiency,” “Phosphorylase A Kinase Deficiency,” and “GSD IX”. We analyzed guidelines and reviewed articles and case reports. We also checked the references list of relevant studies to identify additional missing studies. Data were extracted by two independent authors (E.C. and V.D.N).

### Institutional review board statement

Written informed consent was obtained from the patients for the publication of these cases. Ethics committee approval was not obtained because we reported only three clinical cases, we did not include any identifiable information, and we obtained written consent for publication from the patients’ parents according to the 1964 Declaration of Helsinki and its later amendments or comparable ethical standards. The current retrospective review of patient data did not require ethical approval in accordance with the local guidelines. An ethics statement does not apply to the literature review because this study is based exclusively on published literature. The review was not registered.

## Results

Three patients were diagnosed with GSD IXa all of whom were male. These represent the total number of GSD IXa cases diagnosed and currently followed at our regional metabolic disease center. No additional patients with this specific subtype have been identified to date at our institution. Therefore, the present manuscript includes the complete cohort of GSD IXa cases under our care, ensuring a comprehensive description of the clinical presentation and follow-up data within our center.

### Age at presentation and clinical features at diagnosis

All patients had a clinical onset characterized by episodes of symptomatic ketotic hypoglycemia (Table 1).

In our case series, the age of clinical onset ranged from 10 months to 4 years and 4 months. The median diagnostic delay was 27 months.

**Table 1:** Laboratory, clinical, and instrumental features at clinical onset

Patient	Onset age (y. m.)	Diagnosis age (y. m.)	Elevated liver transaminase levels	Hypoglycemia	Elevated lactic acid	Hyperlipidemia	Stature	Delayed motor development	Liver Ultrasonography
1	4.4	6.6	-	+	-	-	25 pc	-	-
2	1.6	3.9	-	+	-	-	83 pc	-	-
3	0.10	8.3	-	+	-	-	67 pc	-	-

We herein report the clinical history of the three patients, the most relevant biochemical data (Table 2), and the outcome of the genetic investigation (Table 3).

We also report the nutritional characteristics at the first and last dietary evaluation (Tables 4 and 5).

### Case presentation

#### Case 1

Patient 1 came to clinical attention at the age of 4 years and 10 months due to recurrent episodes of symptomatic hypoglycemia consistently occurring in the early morning hours at a frequency of approximately twice per month. These episodes were characterized by pallor, profuse sweating, weakness, hunger, nausea, and vomiting. Symptoms resolved promptly following the intake of sugary beverages.

The patient’s medical history was unremarkable. He was born at term via spontaneous vaginal delivery and exhibited normal postnatal adaptation. No hypoglycemic episodes were reported during the neonatal period and both growth parameters and psychomotor development were within normal limits.

However, at the age of 4 years and 4 months, the patient experienced a hypoglycemic episode during routine blood tests prescribed by the primary care pediatrician. The tests were performed after an overnight fast of approximately 13 h and revealed a blood glucose level of 30 mg/dL, with undetectable insulin levels. Consequently, the patient was admitted to another hospital for further diagnostic evaluation.

The diagnostic investigations included a fasting test that revealed severe hypoglycemia (36 mg/dl) after 12 h, in combination with elevated ketone body levels (3.1 mmol/L), suppressed insulin levels, normal adrenal cortex response, normal ammonia levels, and no metabolic acidosis. Further tests, such as metabolic and abdominal ultrasound, showed normal results.

Magnetic resonance imaging (MRI) of the hypothalamic-pituitary region was performed and revealed no pathological findings.

Due to an insufficient GH response (0.57 ug/l) during hypoglycemia, which was confirmed by a stimulus test with i.v. arginine (GH peak 1.98 ug/l), GH treatment was initiated at 30 mcg/kg/day. However, after starting the GH treatment, the child experienced milder hypoglycemic episodes again in the early morning.

An extensive genetic analysis was conducted as a diagnosis of GH deficiency was unlikely. The results revealed a genetic variant of uncertain significance with maternal inheritance in the PHKA2 gene. A diet plan was established that included extended-release cornstarch (ER-CS) before bedtime and GH treatment was discontinued. One month after discontinuation of GH therapy, a different GH stimulus test (with clonidine by mouth) was performed with a normal result (GH peak 11.6 ug/l).

Since starting the ER-CS diet plan the child has not experienced any further hypoglycemic episodes The family reported satisfaction with the clinical outcome and has returned to a normal daily routine. They now sleep better throughout the night and the child is more active in the morning and throughout the day.

#### Case 2

At 3 years and 9 months, the patient was referred to our center after being hospitalized elsewhere for recurrent hypoglycemia. He was delivered via cesarean section at full term due to breech presentation and experienced normal postnatal adaptation. At 48 h old, he was found to have severe hypoglycemia (8 mg/dl), which was treated with IV glucose. After spending approximately 4 weeks in the hospital, he was discharged without a definitive etiological diagnosis. Following that episode, the patient remained in good health with normal growth and psychomotor development.

At the age of 18 months, the patient experienced a second severe hypoglycemic episode, with a blood glucose level of 18 mg/dL, accompanied by a generalized seizure following a prolonged overnight fast.

Over the next 6 months, two more episodes of symptomatic hypoglycemia occurred upon awakening, which resolved after his taking sugar by mouth. The patient was then admitted to the hospital where a fasting test was performed. The test documented severe hypoglycemia with a low blood sugar level of 27 mg/dl after almost 14 h of fasting. The patient also had a mild elevation of ketone bodies (0.5 mmol/L), ketonuria (+++), suppressed insulin levels, normal adrenal cortex response, and normal ammonia levels. Blood gas analysis and lactate levels were also normal. Abdominal ultrasonography did not reveal any liver-related issues and the brain MRI appeared normal. A comprehensive genetic analysis identified a genetic variant of uncertain significance in the PHKA2 gene inherited maternally (refer to Table 3 for details). The child’s intolerance to CS and ER-Cs resulted in a prescribed diet consisting of six meals, each containing complex carbohydrates. This diet has proven effective in controlling hypoglycemic episodes.

#### Case 3

A 7.5-year-old boy with a history of symptomatic hypoglycemic episodes was admitted to our center for evaluation. He was born by full-term cesarean delivery due to gestational diabetes, which was treated with diet therapy. His postnatal periods and developmental milestones were regular. The first documented hypoglycemic episode occurred at the age of 10 months. Subsequently, at the age of 5 years and 1 month, he underwent an 18-hour fasting test without any episodes of hypoglycemia. Additionally, he underwent continuous glycemic monitoring for 6 days, which showed normal results with a mean blood glucose level of 93 mg/dl and no hypoglycemic episodes.

After experiencing at home recurring symptoms suggestive of fasting hypoglycemia (i.e., sweating, skin paleness, and asthenia), the patient underwent a second fasting test 4 months later. This test detected one episode of hypoglycemia, with a blood glucose level of 35 mg/dl, accompanied by elevated ketonuria, moderate dicarboxylic aciduria, and a slight reduction in free carnitine along with an increased concentration of 3-OH-butyrylcarnitine.

Therefore, dietary advice was given recommending in particular avoidance of prolonged fasting and the intake of complex carbohydrates at every meal.

From the age of 7 years and 4 months, the patient reported frequent daytime and postprandial hypoglycemia, often with symptoms.

As a result, a continuous glucose monitor was repositioned. This monitor detected a few mild symptomatic hypoglycemic episodes with blood glucose levels of 65–67 mg/dl, even after meals, which were resolved by sugar intake.

The patient was admitted to our center where a 180-minute oral glucose tolerance test was performed to exclude postprandial hypoglycemia, with no hypoglycemic episodes observed. Given the persistence of unexplained hypoglycemia, an extensive next-generation sequencing (NGS) panel for hypoglycemia-related genes was subsequently performed which revealed a variant of uncertain significance in the *PHKA2* gene.

**Table 2:** Biochemical features

Blood test	Patient 1		Patient 2		Patient 3	
	First visit	Last visit	First visit	Last visit	First visit	Last visit
Hb (mg/dL)	12.4	12	14.4	14	13.1	13.4
Glucose (mg/dL)	64	86	72	66	72	73
INR	1.12	1.14	1.07	1.02	1.03	1.07
aPTT-ratio	1.07	1.15	1.14	1.18	1.13	1.24
Total cholesterol(mg/dL)	180	157	165	178	228	182
Triglycerides (mg/dL)	53	60	62	64	66	70
ALT (U/L)	27	33	26	18	22	38
AST (U/L)	34	23	43	34	24	31
CPK (U/L)	118	71	104	171	130	124
Basal insulin (µU/mL)	4	4.6	4	4.6	6.9	4.7
IGF1 (ng/mL)	70	269	121	N.A.	157	N.A.
pH	7.37	7.31	7.36	7.32	7.38	7.32
HCO3 (mmol/L)	19	26	26	26	29	24
Lactate (mg/dL)	4	7.8	6.3	8.1	6.5	10

**Table 3:** Molecular analysis of the cohort

Patient	Gene	Variants	Protein	Coding impact	ACMP/AMP classification	Inheritance
1	PHKA2	c.226G > A	p.(Glu76Lys)	Missense	US	M
2	PHKA2	c.2522G > A	p.(Cys841Tyr)	Missense	US	M
3	ENO3 PHKA2	c.121G > A c.2522G > A	p.(Gly347Glu) p.(Cys841Tyr)	Synonymous Missense	P US	M M

Abbreviations: ACMG: American College of Medical Genetics and Genomics, AMP: aAssociation for Molecular Pathology, P: pathogenetic, LP: likely pathogenetic, A: absent, US: of uncertain significance, LB: likely benign, B: benign [13]

**Table 4:** Dietary intake at first visit

Dietary intake at first visit	Patient 1	Patient 2	Patient 3
Age (Y, M)	6,.5	4.10	7.7
Weight (kg)	22.8	23.2	30.8
Carbohydrate %	51	53	46
Simple sugars %	14	20	13
Lipid %	33	33	40
Protein %	16	14	14
Total calories	1630	1590	1650
Calories/kg	71	68	54
Starch	No	No	No

**Table 5:** Dietary intake at the last visit

Dietary intake at last visit	Patient 1	Patient 2	Patient 3
Age (Y, M)	7,.2	6.6	9.5
Weight (kg)	24	29	38,.6
Carbohydrate %	50	42	54
Simple sugars %	15	13	9
Lipid %	34	43	31
Protein %	16	15	15
Total calories	1790	1970	2200
Calories/kg	74	68	57
Raw/modified corn starch g/die	24	No	53
Raw/modified corn starch g/kg/dose	1 g/kg/dose (single dose)	No	0.5 g/kg/dose (3 doses)
Significant weight gain	No	No	Yes (5 kg in 6 months)

## Discussion

We present three cases of persistent and pronounced fasting-induced ketotic hypoglycemia, in the absence of other clinical signs or symptoms, which led to the diagnosis of an underlying metabolic disorder.

Although simple ketotic hypoglycemia, typically occurring between 18 months and 5 years of age and usually resolving spontaneously by age 6, is the most common cause of hypoglycemia in children, the presence of additional signs such as primary hepatomegaly may suggest an alternative underlying pathological condition [14]. In our experience, symptomatic ketotic hypoglycemia was not found to be associated with any additional clinical signs, which constitutes a noteworthy aspect of our limited case series.

The age of onset of the disease overlaps with that of uncomplicated ketotic hypoglycemia; however, if episodes persist and recur beyond the age of 5, this may serve as a clear indication for further diagnostic evaluation.

Newborn screening programs in Italy, where comprehensive testing is implemented, systematically evaluate various conditions that may predispose to hypoglycemia between 48 and 72 h of life [15].

Among the conditions that can lead to hypoglycemia and present with early onset acute metabolic decompensation are defects in beta-oxidation and galactosemia, which highlight the importance of early diagnosis to prevent potential complications [15, 16]. All our patients have undergone expanded newborn screening, which has given us a significant advantage in ruling out several severe metabolic conditions. Regarding our diagnosis of GSD IXa, Burwinkel et al. have described the high clinical variability of this condition in terms of fasting hypoglycemia associated with severe clinical presentations [17].

Since that paper, much progress has been made in the research into and knowledge about this type of GSD, culminating in a recent first comprehensive literature review that questions the pathogenicity or benignity of this condition [8].

Although hepatomegaly was present in 93.2% of GSD IXa patients, the presenting symptoms were considered benign and tended to improve with age [8].

A 2020 Italian study found liver involvement in 7/9 GSD IXa patients with available ultrasound reports [18].

Tagliaferri et al. reported on 23 Italian patients with GSD-IX of whom 17 were males affected by GSD-IXa. The study found that 20 of the 23 patients had hepatomegaly, indicating a high prevalence of this symptom in GSD-IXa patients. Another study from Canada’s largest metabolic center also recommends close monitoring of all GSD-IXa patients due to long-term liver complications, as reported by Roscher in 2014. In an additional case series of 11 patients with GCS-IXa, hepatomegaly was found in 67% of cases, while short stature and hypoglycemia were reported in 22% and 17% of cases, respectively. In a recent systematic literature review, patients with GSD IXa and fasting hypoglycemia numbered 28.8%, while hepatomegaly was found in 74.8% of cases [6].

It is important to note that many existing studies on GSD-IX refer to populations with diagnoses made before the latest significant advances in genetic testing, such as NGS gene panel testing or exome. In these cases, notable clinical signs were often the main diagnostic clue, leading to more severe clinical phenotypes and a more significant diagnostic delay. It is possible that our ability to diagnose our patients who do not have hepatomegaly, in contrast to previous reports on GSD IXa, is due to increased knowledge of the condition and the more widespread use of genetic panels.

In terms of treatment, a nutrition plan is essential to improve metabolic control and prevent complications, as advised by the American College of Medical Genetics and Genomics (ACMG) in 2019 [13]. This plan involves frequent feeding with complex carbohydrates and protein during the daytime as an alternative source of glucose [5].

At night, before bedtime, children should consume CS or ER-CS, Glycosade^®^, at a dosage of 1 g/kg to maintain euglycemia for 4–8 hours [5].

CG is commonly prescribed beyond 12 months of age (the age at which the digestive enzyme amylase appears to be fully active), even though this treatment has been introduced in some patients as early as at 6 months of age [5].

ER-CS has proven beneficial in children over 5 years old, most of all in extending the control of glycemia during the night [19, 20].

The effectiveness of uncooked CS lasts almost 4 h. Therefore, patients require at least one feeding in the middle of the night, a problem solved using ER-CS [20, 21]. Data from the first randomized, double-blind, cross-over, multicenter international trial were published in 2024 demonstrating both the efficacy and tolerability of Glycosade^®^ in a large cohort of patients with hepatic GSD [22].

The benefits of CS are highly recommended for the GSD type I population, a more severe pathology requiring more frequent doses throughout the day. At the same time, the description of the IX-affected case series is much rarer.

In the 2020 Italian study, all but one patient with GSD IXa was on uncooked CS therapy at the last follow-up, while no patient developed clinical signs of advanced chronic liver disease, synthetic liver failure, focal ultrasound changes, or portal hypertension [18].

Our case series shows that all patients have achieved reasonable glycemic control. Patient 1 only follows a diet with more frequent and fractionated meals, patient 2 takes CS, and patient 3 takes ER-CS. This demonstrates the significant variability of the phenotypic spectrum, even within the same pathology and mutation (patients 2 and 3). A recent literature review sought to establish phenotype-genotype correlations for the genes implicated in the four subtypes of GSD IX [23].

Significant weight gain is one of the most common complications of CS use. However, in our experience, only patient 3 showed a critical weight gain after a follow-up of 1 year. This is not surprising as this patient is the only one taking ER-CS three times a day. In addition, weight gain was reported especially in those diagnosed before the age of 2 years in other Italian case series, likely due to a more hypercaloric and hyperglucidic diet [24]. Patient 2 had poor tolerance to both CS and ER-CS, resulting in abdominal bloating. According to the 2019 guidelines, it is recommended to monitor blood glucose and ketone levels during significant changes in diet and at times of stress. Additionally, a home device for continuous glucose monitoring can help manage various types of GSD [25].

In our case series, establishment of an early nutrition plan could enable us to prevent primary manifestations (ketotic hypoglycemia) and secondary complications (hepatomegaly, short stature, and delayed puberty).

Another important observation as regards our patients is related to the genetic investigation of the patients. The mutations we identified in the PHKA2 gene, the variants c.226G > A and c.2522G > A, are both described as VUS by the American College of Medical Genetics and Genomics, whereas in our case series, the variants were found to be causative of symptomatology. Since this disease has an X-recessive trait, the higher phenotypic expressibility in male patients is known. The clinical symptoms of these patients and the excellent therapeutic response cause us to hypothesize that the clinical manifestations can be defined as pathogenic for GSD IXa.

The natural history and long-term outcome of GSD IXa are rarely reported. It is commonly believed that many biochemical and clinical anomalies gradually disappear with age and that most adult patients are asymptomatic [26].

## Limitations

We acknowledge that our study has some limitations. Although our population sample was significant, it was relatively small. Additionally, we collected the patients’ data retrospectively from their first to last evaluations. Still, not all data were available, this primarily due to the examinations conducted before the patients arrived at our center.

## Conclusion

Our clinical cases have shown that patients with GSX IXa present only with hypoglycemia without any liver abnormalities or other significant symptoms. Therefore, if a patient experiences recurrent episodes of ketotic hypoglycemia and other endocrinological causes that have been ruled out, it is crucial to consider genetic testing for GSX IXa. A timely diagnosis can help establish an appropriate diet plan and prevent complications.

## Data Availability

The original contributions presented in the study are included in the article; further inquiries can be directed to the corresponding author.
